# An Ultrashort Wavelength Multi/Demultiplexer via Rectangular Liquid-Infiltrated Dual-Core Polymer Optical Fiber

**DOI:** 10.3390/ma12101709

**Published:** 2019-05-26

**Authors:** Qiang Xu, Kang Li, Nigel Copner, Shebao Lin

**Affiliations:** 1 College of Physics and Optoelectronic Technology, Baoji University of Arts and Sciences, Baoji 721016, China; linshebao@163.com; 2 Wireless and Optoelectronics Research and Innovation Centre, Faculty of Computing, Engineering and Science, University of South Wales, Cardiff CF37 1DL, UK; nigel.copner@southwales.ac.uk; 3 Foshan Huikang Optoelectronics Ltd., B block, Sino-European Center, Foshan 528315, China

**Keywords:** polymer optical fiber, couplers, coupling length, birefringence

## Abstract

We propose a rectangular liquid-infiltrated dual-core polymer optical fiber (POF) for short-range communication systems by the beam propagation method (BPM). The POF multi/demultiplexer (MUX/DEMUX) at the wavelengths of 0.52/0.65-μm, 0.57/0.65-μm, and 0.52/0.57-μm are devised. The simulation results demonstrate that the ultrashort length of three ultrashort POF couplers are 183.6 μm, 288 μm, and 799.5 μm. Compared with the conventional optical fiber couplers, these results could have significant applications in the miniaturization of optical devices for visible light communication.

## 1. Introduction

In recent years, with the rapid expansion of optical communication technology, application demands of users for optical transmission networks and systems are growing exponentially. In order to relieve the huge pressure of bandwidth for an optical communication network and system, all kinds of measures have been proposed to increase the capacity of optical networks [1,2,3,4,5,6,7,8]. Wavelength division multiplexing (WDM) is a key technology in advanced optical communication networks. The use of WDM technology not only significantly increases the capacity of the existing optical communication networks without increasing the number of fibers, but also possesses advantages in flexible services, network provision, and network management [9]. The wavelength division multi/demultiplexer (MUX/DEMUX) is an important passive component in the WDM system, which splits/combines lights with different wavelengths into different outputs [10]. All sorts of wavelength MUX/DEMUXs using silica-PLC [2], InGaAs/InP avalanched photodiodes [11], Chirped fiber Bragg grating [12], 2D/3D photonic crystal [13,14], polymer photonic structure [15], and photonic crystal fiber [4] have been demonstrated. At present, the wavelengths of most wavelength division MUX/DEMUXs are located in near-infrared wavelengths [2,4,11,12,13,14,15]. There are very few wavelength division MUX/DEMUXs in the visible wavelengths. Therefore, the design and application of such optical devices in the visible wavelengths are of great significance to develop the visible short-distance communications systems.

Photonic crystal fibers (PCFs) are a special class of optical fibers characterized by a periodical arrangement of microcapillaries that form the fiber’s cladding around a solid or hollow defect core [16,17,18]. Since then, various fiber types such as honeycomb PCF [19], triangular PCF [20], rectangular PCF [21,22,23,24], D-shape PCF [25], side-polished PCF [26], and metal-coated PCF [27] have been demonstrated and applied in the fields of pharmaceutical drug testing, astronomy, communication, and biomedical engineering and sensing [28,29,30,31,32,33,34,35,36,37]. Compared with conventional optical fibers, the unique features of PCFs, such as flexibility of design, endless single-mode wavelength [21], high birefringence [22,23,24], and adjustable dispersion and nonlinearity [38,39], can be obtained by manipulating lattice period, air hole size and shape, refractive index of the materials, and type of lattice. In general, specific categories of PCF include photonic bandgap fibers and index-guiding holey fibers. In this article, we mainly focus on index-guiding holey fibers. The PCFs filled with materials have attracted great interest because PCFs could provide excellent properties by filling different functional materials into the air holes [40,41,42,43]. The PCFs present high-quality microfluidic channels that can be controllably filled with ultrasmall volumes of analytes [44], such as benzene [45,46], glycerin [47], water [48], alcohol [49], and nematic liquid crystal [50]. High birefringence can not only maintain the linear polarization state in the fiber but also increase the difference in coupling length of *x*-polarized mode and *y*-polarized mode of PCF. In general, high birefringence of fibers can be gained by introducing asymmetric defect structure such as air holes of different shapes and sizes and asymmetric core [51,52,53]. Another kind of high birefringence structure is the rectangular lattice PCF [21,22,23]. Ferrando et al. found that all polarization guided modes doublets are degenerate in practice in honeycomb and triangular PCF by using precise full-vector calculations, indicating that both geometric structures have negligible anisotropy [18,19,20]. The rectangular lattice is potentially more anisotropic than the triangular and honeycomb lattices. Therefore, based on the above advantages of PCFs, we design a rectangular liquid-filled photonic crystal fiber with high birefringence, which combines the liquid filling, introduction of asymmetric defect structure, and high anisotropy. 

Polymer optical fibers (POFs) have a well-established use as a low-cost alternative to silica fiber in short-range communications systems, for example, digital home appliance interfaces, home and car network, and traffic control application, where the attenuation of polymers is very low [54,55]. The cost advantage mainly comes from the ease of installation of the POF fiber, which is simple to cleave and connect and is highly flexible, even with the standard 1mm diameter [56]. A variety of polymers can be used as POFs, such as polymethyl-methacrylate (PMMA), polyethylene, cyclic-olefin copolymer (COC), cyclo-olefin polymer (COP), and Teflon [57,58]. The PMMA has very low attenuation at 520 nm, 570 nm, and 650 nm [59,60,61]. In addition to the capillary stacking technology traditionally used for glass PCF, POFs can be made using techniques such as extrusion, bulk polymerization process, drilling–stretching, and injection molding [62,63,64]. The advantage of such techniques is that different cross-sections can be obtained in the preform, with arbitrary shape and size of the holes [65]. These characteristics of POFs have opened possibilities for the visible wavelength multi/demultiplexers in short-range communication systems. 

In this paper, a numerical analysis of a novel rectangular liquid-infiltrated dual-core POF is presented using a vector beam propagation method (BPM) [66,67,68]. The ultrashort POF couplers for 0.52/0.65-μm, 0.57/0.65-μm, and 0.52/0.57-μm wavelength MUX/DEMUX are devised for WDM applications in the visible wavelengths. Besides the great performance, compared with the reported results [25,30,31,32,42,51], another advantage of the proposed structure is that it is simplified so that only the size of filling core and cladding air holes need to be adjusted to achieve the coupler behavior with ultrashort length. Therefore, the proposed couplers possess easy fabrication merit.

## 2. Design Principle and Theoretical Modeling

**Figure 1:**
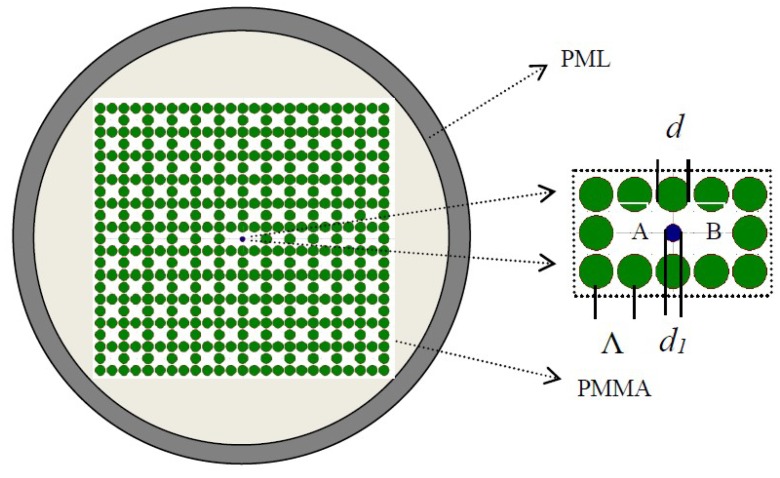
Cross-section of the proposed polymer optical fiber. The enlarged view of the core region is shown in the illustration.

Figure 1 demonstrates a model in which the core region (see the enlarged view of Figure 1) and the cladding region are organized in rectangular formation across the PMMA backdrop. In contrast to a glass optical fiber, the PMMA is a popular material for optical fibers due to the low polymerization temperature, low cost, high transparency, and high mechanical flexibility [57,58]. With the cladding, the green air holes have a diameter of *d*. In the core region, *d_1_* is the diameter of the blue air hole filled with benzene (*n* = 1.366). Benzene is a highly toxic carcinogen. How to quickly and easily detect benzene in the environment and food is very important [69]. Therefore, the sensing characteristics of polymer devices based on filled benzene will be considered in future work. The distance of hole to hole can be expressed as period Λ, the air-filling ratio is *d/*Λ. The refractive index of background material is set as 1.49.

The vector wave equation, which is the basis of BPM [66,67,68], can be expressed by

(1) $$\nabla^{2}E+k^{2}E=0$$

(2) $$\nabla^{2}H+k^{2}H=0$$

where $k\equiv \omega \sqrt{\mu \varepsilon }$. These two equations are known as the Helmholtz equations.

The electric field *E*(*x*, *y*, *z*) can be separated into two parts: the fast change term of exp(-*ikn_0_z*) and the envelope term of *ϕ*(*x*, *y*, *z*) of slow change in the axial direction, *n_0_* is a refractive index in the cladding. Then, *E*(*x*, *y*, *z*) is stated as 

(3) $$E(x,y,z)=\phi (x,y,z)\exp(ikn_{0}z)$$

Substituting Equation (3) in Equation (1) results in

(4) $$\nabla^{2}\phi -2ikn_{0}\frac{\partial \phi }{\partial z}+k^{2}(n^{2}-n_{0}^{2})=0$$

where *n* is a refractive index in the fiber core.

The relative refractive index is an important parameter to describe the constrained optical field capability of fiber which is expressed as

(5) $$\Delta =\frac{n^{2}-n_{{}_{0}}^{2}}{2n_{{}_{0}}^{2}}$$

when Δ<0.01, $\Delta ≅\frac{n-n_{0}}{n_{0}}$ is called the weakly guiding condition.

Assuming the weakly guiding condition, we can approximate $n^{2}-n_{0}^{2}≅2_{n_{0}}(n-n_{0})$. Then Equation (4) can be rewritten as 

(6) $$\frac{\partial \phi }{\partial z}=-i\frac{1}{2kn_{0}}\nabla^{2}\phi +jk(n-n_{0})\phi$$

A similar expression can be written for **H**. We find that $n\neq n_{0}$ if the fields vary in the transverse direction to propagation. Light propagation in various kinds of waveguides can be analyzed by the above method.

The birefringence is an important index to evaluate the performance of polarization maintaining, which is expressed as [51,52,53,70]

(7) $$B=\left|n_{x}-n_{y}\right|$$

where *n_x_* and *n_y_* represent the effective refractive index of *x*-polarization and *y*-polarization, respectively.

There are four modes of dual-core PCF on the basis of the principle of coupling mode, namely, even-mode of *x*-polarization, odd-mode of *x*-polarization, even-mode of *y*-polarization, odd-mode of *y*-polarization. The coupling length can be defined as [4]

(8) $$L_{c}^{x,y}=\frac{\lambda }{2\left|n_{even,\lambda }^{x,y}-n_{odd,\lambda }^{x,y}\right|}$$

where $n_{even}^{x,y},n_{odd}^{x,y}$ denote the effective indexes of even-mode of *x*-polarization, odd-mode of *x*-polarization, even-mode of *y*-polarization, odd-mode of *y*-polarization, respectively. 

When *L_λ1_* and *L_λ2_* satisfy the following Equation (9) or (10), a polymer coupler can separate two wavelengths λ_1_ and λ_2_ transmitted in a core [4].

(9) $$L_{\lambda_{1}}:L_{\lambda_{2}}=\mathrm{even}:\mathrm{odd}$$

or

(10) $$L_{\lambda_{1}}:L_{\lambda_{2}}=\mathrm{odd}:\mathrm{even}$$

Assuming that the incident power is emitted to a certain core, the output power of *x*- or *y-*polarized light in the core can be expressed [71].

(11) $$P_{out}^{x,y}=P_{in}^{x,y}\cos^{2}(\frac{\pi }{2}\cdot \frac{z}{L_{c}^{x,y}})$$

where the transmission distance is denoted by *z*. 

The confinement loss of PCF is calculated from the imaginary part of the effective refractive index, using the following equation [36],

(12) Confinement loss=8.686k0Im(neff)[dB/m]

## 3. Simulated Results and Analysis

**Figure 2:**
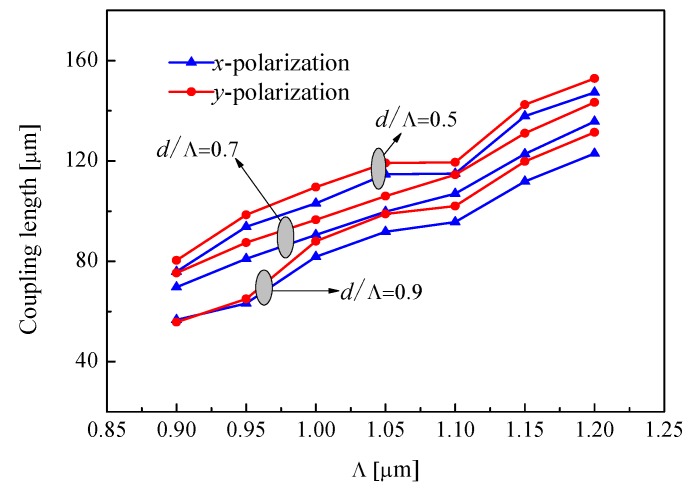
Period Λ dependence of the coupling lengths for different air-filling ratio *d*/Λ.

First, we analyze the coupling lengths as a function of period Λ for different air-filling ratio *d*/Λ, where *d*_1_ = 0.4 μm as shown in Figure 2. It is observed that the coupling length is increased when period Λ is increased for a constant air-filling ratio *d*/Λ. Moreover, the coupling length of *y*-polarization is higher than the coupling length of *x*-polarization. Since the *x*-axis is parallel to the core A and core B, the coupling length of the *y*-polarization is smaller than that of the *x*-polarization. Furthermore, we can clearly see that coupling length is decreased when air-filling ratio *d*/Λ is increased for the same value of period Λ. This is because the restriction of the outer cladding to the light wave is enhanced as the air-filling ratio increases. For the coupler with excellent performance, not only the strong coupling effect between core A and core B but also the good extinction ratio should be considered [4]. Based on the above considerations, we decided to use the *y*-polarization for the polymer optical fiber couplers.

**Figure 3:**
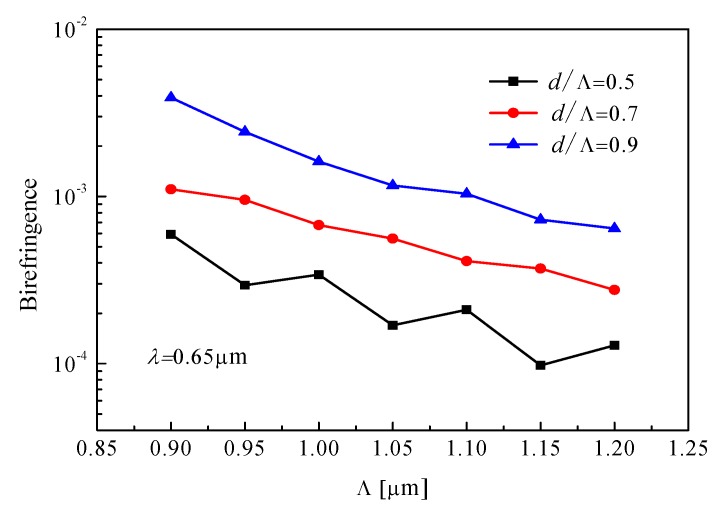
Period Λ dependence of the birefringence for different air-filling ratio *d*/Λ.

High birefringence can not only maintain the linear polarization state in the fiber but also increase the difference in coupling length of *x*-polarized mode and *y*-polarized mode of PCF. Figure 3 shows the birefringence as a function of period Λ. We found that the birefringence of PCF increases with the increase of air-filling ratio *d*/Λ, which results in stronger coupling strength between the two cores for a shorter coupling length of the polymer optical fiber. Based on the high birefringence of the fiber, we chose the air-filling ratio of 0.9. 

**Figure 4:**
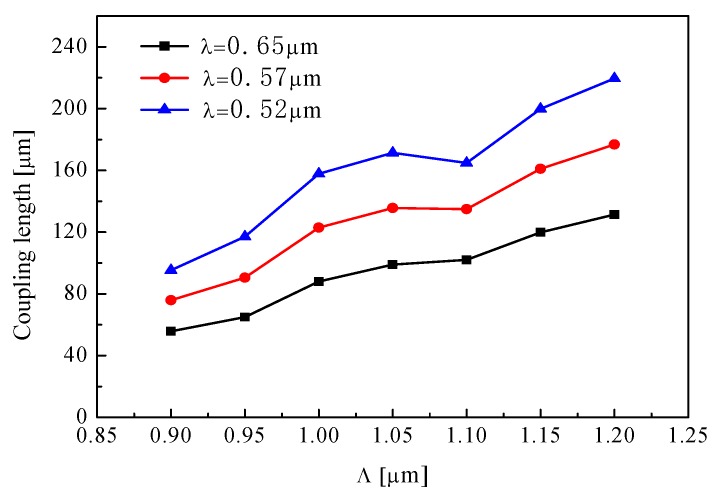
Period Λ dependence of the coupling length for different wavelength.

Additionally, when *d*_1_ = 0.4 μm, *d/*Λ = 0.9, the coupling length of *y*-polarized mode is shown as a function of period Λ in Figure 4, in which it is observed that the coupling length is increased if period Λ is increased. As the period increases, the coupling between the cores becomes weaker. Meanwhile, the coupling length of *y*-polarization decreases with increasing operating wavelength.

**Figure 5:**
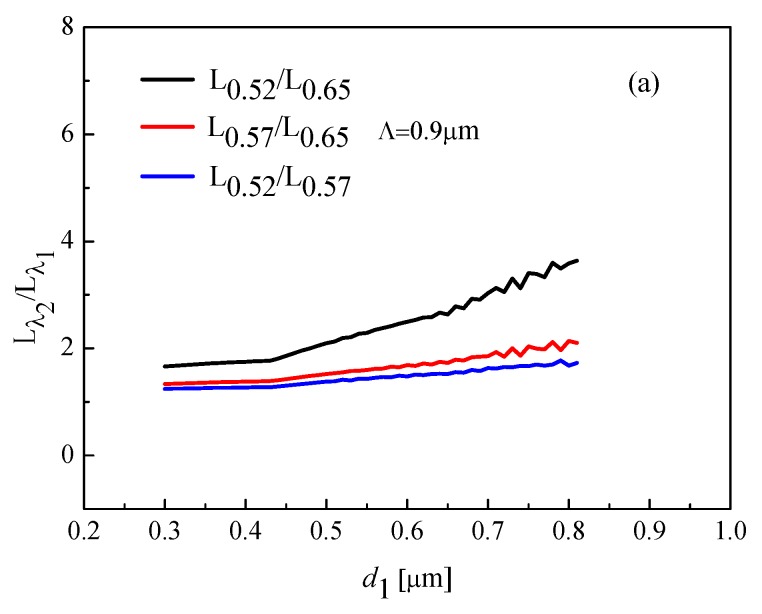

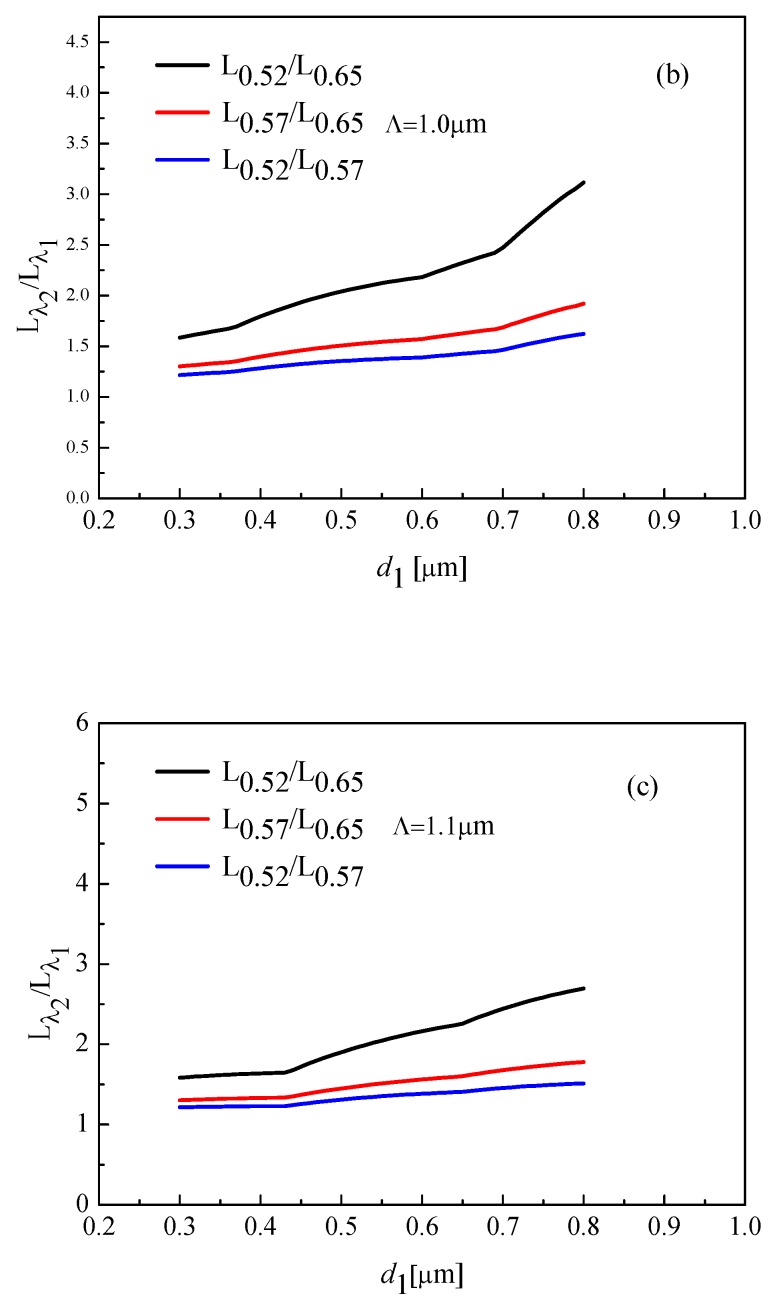
*d_1_* dependence of the different coupling ratios, (**a**) when *d/*Λ = 0.9, (**b**) when *d/*Λ = 1.0, and (**c**) when *d/*Λ = 1.1.

Figure 5 shows coupling ratio *L_λ2_*:*L_λ1_* by changing *d*_1_ from 0.3 to 0.8 for different period Λ and *d/*Λ = 0.9. The one wavelength is 0.52 μm and 0.57 μm, and the other wavelength is 0.57 μm or 0.65 μm. Therefore, we obtain three couplers, named coupler 1, 2, and 3. In order to obtain an excellent coupling effect, we choose *d/*Λ = 0.9, Λ *=* 0.9 μm as the optimal parameters. When *d/*Λ = 0.9, Λ *=* 0.9 μm, and *d*_1_ = 0.4 μm, *L_0.57_*: *L_0.65_* and *L_0.52_*:*L_0.57_* for couplers cannot satisfy Equation (9) or (10); *L_0.52_*:*L_0.65_* for coupler is 7:4. In order to obtain the ultrashort coupler, we analyze the influence of parameter d_1_ on the length of the coupler through Figure 5c. Finally, the couplers 1 to 3 are *L_0.52_*:*L_0.65_* = 2:1, *L_0.57_*:*L_0.65_* = 3:2, and *L_0.52_*:*L_0.57_* = 3:2, respectively.

Table 1 shows the optimal parameters of three different wavelength couplers. We can clearly see that the length of coupler 1 is shorter than coupler 2 and 3. This phenomenon is probably related to the difference between the coupling length at 0.65 μm and 0.52 μm. Meanwhile, the length of couplers is much shorter than optical couplers in the References [4,72,73]. The main reasons for the ultrashort coupler are related to the design of fiber structure (rectangular lattice structure), the selection of background materials (PMMA), and the filling of functional materials (benzene).

**Figure 6:**
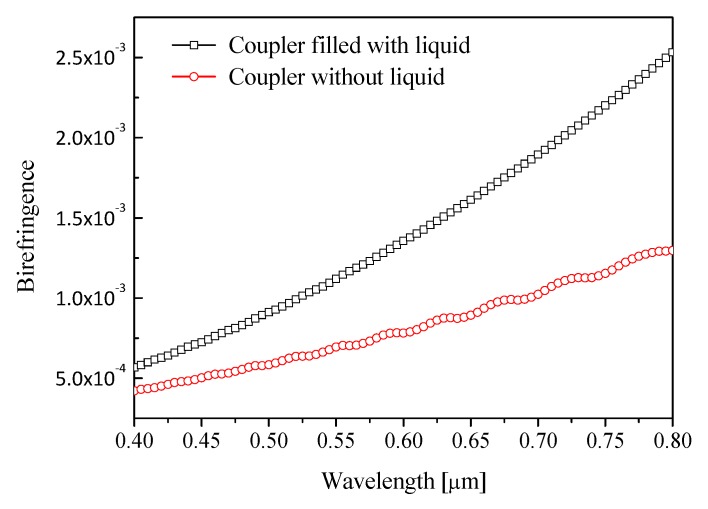
Birefringence as a function of filling material.

**Figure 7:**
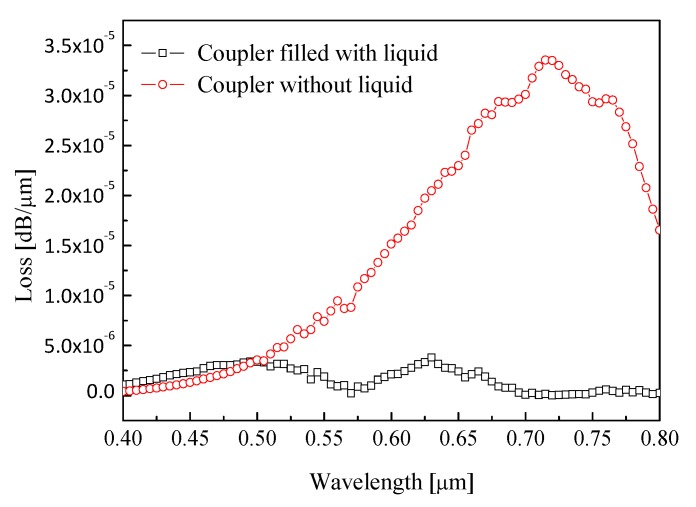
Confinement loss as a function of filling material.

In order to analyze the influence of liquid filling on the transmission performance of couplers, we study the relationship between liquid filling and birefringence and confinement loss of couplers. Figure 6 shows the relationship between the birefringence and filling material for *d*_1_ = 0.48 μm, Λ = 0.9 μm, and *d/*Λ = 0.9. It is observed that birefringence of coupler filled with liquid is higher than birefringence of coupler without liquid. Figure 7 shows the variation of confinement loss with filling material for *d*_1_ = 0.48 μm, Λ = 0.9 μm, and *d/*Λ = 0.9. It can be seen that the confinement loss of the coupler without liquid is higher the confinement loss of the coupler with filled liquid. Therefore, the coupler with filled liquid has lower confinement loss and higher birefringence than the coupler without liquid.

**Figure 8:**
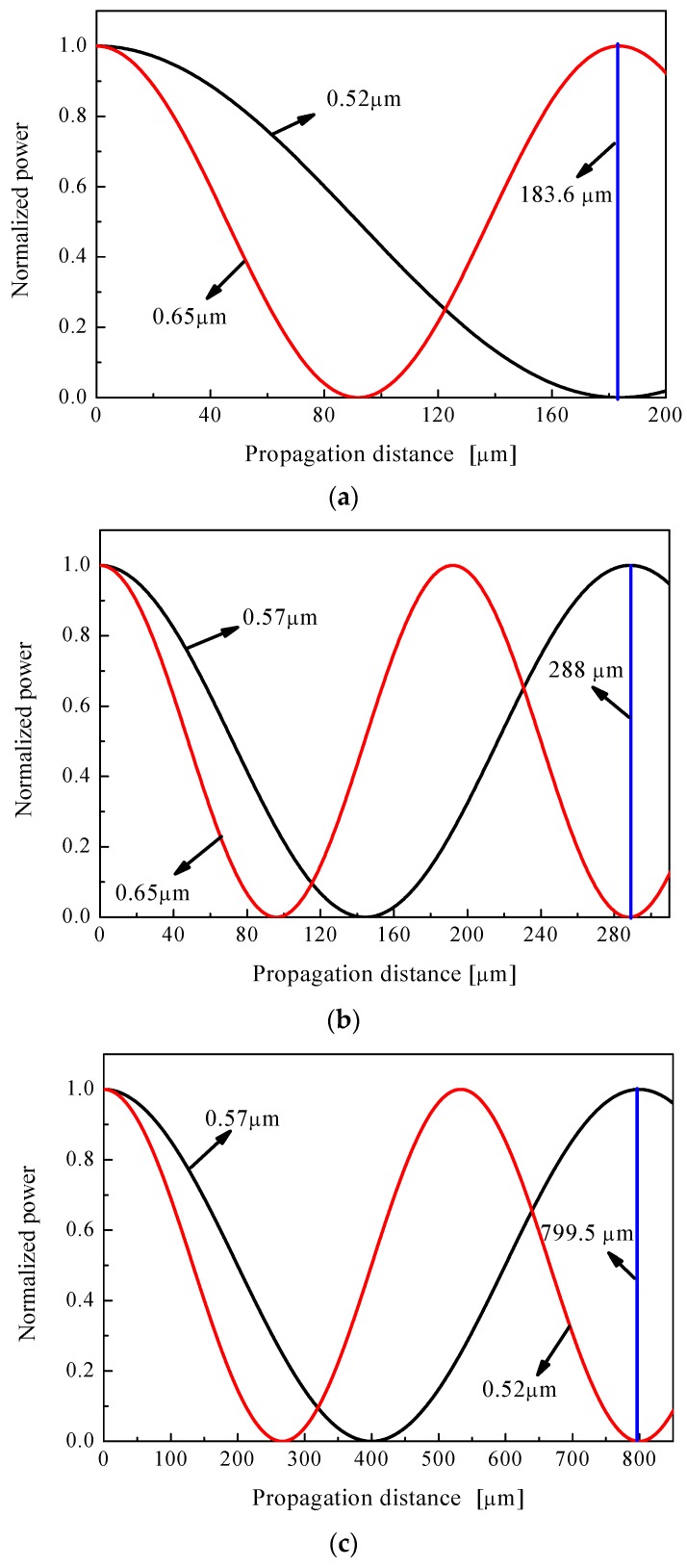
The propagation distance dependence of the normalized power for the three couplers, (**a**) at the wavelength of 0.52/0.65-μm, (**b**) at the wavelength of 0.57/0.65-μm, and (**c**) at the wavelength of 0.52/0.57-μm.

We also demonstrate that three couplers can separate λ_1_ and λ_2_ according to the simulation results by BPM. The fundamental modes of *y*-direction at λ_1_ and λ_2_ are imported into the core A or core B in Figure 1. Figure 8 shows the propagation distance dependence of the normalized power. We observed that the separation of two wavelengths of λ_1_ and λ_2_ for couplers 1 to 3 are achieved at the distances of 183.6 μm, 288 μm, and 799.5 μm, respectively shown as the blue line in Figure 8. Obviously, the three polymer optical fiber couplers can operate as wavelength MUX/DEMUX at the wavelength of 0.52/0.65-μm, 0.57/0.65-μm, and 0.52/0.57-μm, respectively.

**Figure 9:**
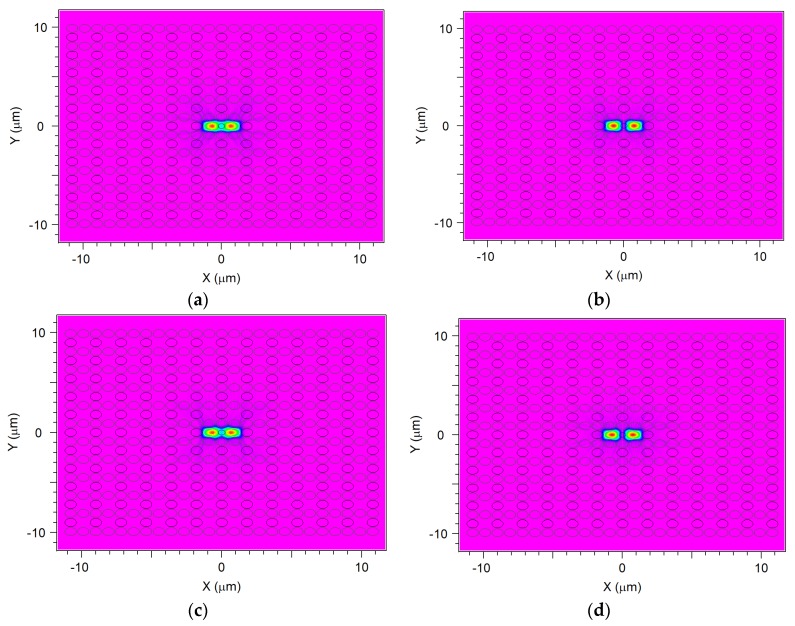
(**a**) Even-mode of *x*-direction, (**b**) odd-mode of *x*-direction, (**c**) even-mode of *y*-direction, (**d**) odd-mode of *y*-direction for coupler.

Figure 9 shows odd- and even-mode of *x*-polarization and *y*-polarization for the coupler, when *d*_1_ = 0.48 μm, Λ = 0.9 μm, and *d/*Λ = 0.9. It shows the mode-field distribution of the odd- and even- mode in two vertical directions. Moreover, the difference propagation constants and phase difference change of odd- and even-mode in transmission results in a power transfer between two cores [74]. 

## 4. Conclusions

Three ultrashort couplers based on rectangular liquid-infiltrated POF have been demonstrated by BPM. The POF couplers for 0.52/0.65-μm, 0.57/0.65-μm, and 0.52/0.57-μm wavelength multi/demultiplexer (MUX/DEMUX) are designed by manipulating structural parameters. The numerical results demonstrate that the lengths of three ultrashort POF couplers are 183.6 μm, 288 μm, and 799.5 μm for the wavelength multiplexing. Compared with the conventional optical fiber couplers, the POF couplers in the visible wavelengths have an ultrashort length, which is important for the application in the miniaturization of optical devices in short-range telecommunication networks [75,76].

## Figures and Tables

**Table 1:** Design parameters of the polymer optical fiber couplers.

Parameters	Couplers					
	1	2	3	4 (Reference [72])	5 (Reference [73])	6 (Reference [4])
λ_1_ [μm]	0.65	0.65	0.57	1.55	1.55	1.55
λ_2_ [μm]	0.52	0.57	0.52	0.85/0.98/1.3/1.48	0.98/1.31	0.85/0.98/1.3/1.48
Λ [μm]	0.9	0.9	0.9	-	-	-
*d*/Λ	0.9	0.9	0.9	-	-	-
*d_1_* [μm]	0.48/0.4	0.49	0.62	-	-	-
Length [μm]	183.6/452.3	288	799.5	4168	2475	1178/418/712/2284
